# Editorial for the Special Issue “Emerging Topics in Population Genetics and Molecular Anthropology: Integrating Genomes, Populations, and the Human Past”

**DOI:** 10.3390/genes17091158

**Published:** 2026-09-21

**Authors:** Jelena Šarac, Dubravka Havaš Auguštin

**Affiliations:** 1 Centre for Applied Bioanthropology, Institute for Anthropological Research, 10000 Zagreb, Croatia; 2 Faculty of Biotechnology and Drug Development, University of Rijeka, 51000 Rijeka, Croatia

Population genetics and molecular anthropology have undergone remarkable methodological and conceptual development over the past two decades. The increasing accessibility of high-throughput sequencing, improvements in ancient DNA (aDNA) recovery, genome-wide approaches to population structure, and increasingly sophisticated computational methods have transformed our ability to reconstruct human demographic history and investigate genetic diversity across both space and time. At the same time, the boundaries between population genetics, molecular anthropology, bioarchaeology, forensic genetics, and evolutionary anthropology have become less distinct. Human genetic variation is no longer studied independently of archaeological, historical, morphological, or demographic information, but rather as one component of a broader interdisciplinary reconstruction of human populations.

The Special Issue “Emerging Topics in Population Genetics and Molecular Anthropology” was developed as a response to this rapidly developing scientific landscape. Its aim was to bring together studies that show how genetic and genomic approaches can contribute to questions concerning population structure and history, human evolution, archeogenetics, forensic and biological anthropology, and patterns of human genetic diversity. Together, these contributions highlight the field’s growing analytical capabilities and the continued need to interpret molecular evidence alongside historical, archaeological, and anthropological evidence.

One important theme represented in this Special Issue is the characterization of genetic structure in contemporary and historically distinctive populations. Szabó et al. [1] investigated the genome-wide autosomal structure of the Sekler population of Transylvania, a Hungarian-speaking population whose demographic history and geographic position make it particularly informative for studies of Central and Eastern European population history. Using high-density autosomal data and combining allele-frequency- and haplotype-based approaches, the authors demonstrated a relatively coherent genetic structure and close relationships with other Hungarian populations. The study illustrates the value of genome-wide data for resolving population relationships that may not be apparent from linguistic, historical, or cultural classifications alone. More broadly, it highlights an important principle of molecular anthropology: cultural identity, language, and genetic history represent related but distinct dimensions of population history and should therefore be interpreted jointly rather than assumed to coincide.

A complementary temporal perspective is provided by Marjanović et al. [2], who investigated maternal and paternal population history in Croatia by integrating 19 newly generated ancient genomes with 285 previously published ancient genomes spanning the Neolithic, Bronze and Iron Ages, Antiquity, and the medieval period, and comparing these data with the contemporary Croatian population. The study reveals a predominantly European maternal genetic profile and, when ancient and modern data are considered together, points to different long-term dynamics of maternal and paternal lineages. Mitochondrial lineages show comparatively stronger persistence through time, whereas Y-chromosomal variation indicates greater temporal differentiation, including substantial changes in the frequencies of certain lineages between prehistoric and modern populations. These findings reinforce the importance of examining maternal and paternal demographic histories separately and demonstrate how sex-specific patterns of migration, population turnover, and social organization may leave different signatures in the genetic record. Southeastern Europe, positioned between Central Europe, the Mediterranean, and the Near East, remains particularly informative for investigating repeated demographic movements across European prehistory and history.

The contribution by Rubini et al. [3] broadens the chronological scope of the Special Issue considerably by examining hominin remains from Guattari Cave in central Italy. Detailed morphometric and comparative analyses of cranial, dental, and postcranial remains dated to approximately 66–65 ka revealed substantial morpho-anatomical variability, including both primitive and derived characteristics and affinities with different groups within the broader Pleistocene hominin record. Rather than presenting past hominin populations as morphologically homogeneous entities, these findings emphasize variability and the potentially complex evolutionary relationships among Pleistocene populations. The study also demonstrates the continued importance of morphological evidence within an increasingly molecular field. Genomic data have profoundly changed the study of human evolution, but fossils, archaeological context, chronology, and morphology remain indispensable for understanding biological variation in populations for which genetic material may be unavailable or poorly preserved. The most informative reconstructions will increasingly emerge from the integration of these different sources of evidence.

Ancient genomics also offers an exceptional opportunity to investigate populations that are poorly represented in the historical or genetic record. Pallarés-Viña et al. [4] provide an important example through their genomic investigation of individuals from the Roquetes necropolis in Tàrrega, Catalonia, associated archaeologically and historically with the anti-Jewish violence of 1348. Genome-wide and uniparental analyses revealed affinities with ancient and contemporary Jewish populations, while ancestry modelling supported contributions related to both Eastern Mediterranean and medieval Iberian sources. Importantly, this work provides the first genomic information from medieval Iberian Jewish individuals and therefore fills a notable gap in European archaeogenomics. Such studies of historically identifiable communities and victims of violence underline the ethical responsibilities associated with aDNA research. Genetic findings must be interpreted within their historical context and with particular care when they intersect with questions of ancestry, community identity, persecution, and cultural affiliation.

The interface between molecular and traditional biological anthropology is further explored by Alves-Cardoso et al. [5]. In a nineteenth-century Portuguese skeletal collection, the authors compared conventional morphological sex estimation with genetic sex determination using aDNA. Analysis of 37 skeletons revealed discordance between the two approaches, demonstrating limitations associated with the application of generalized osteological standards across populations. The results emphasize the effects of population-specific patterns of sexual dimorphism and support the use of molecular approaches as an independent means of validating biological profiles derived from skeletal morphology. This contribution has implications extending beyond the particular collection investigated. Biological and forensic anthropology have historically relied on reference collections and classification standards developed from specific populations. Population variation, secular change, environmental influences, and differences in sexual dimorphism can all reduce the transferability of such methods. Molecular validation therefore provides an important route toward identifying these limitations and developing more robust, population-aware approaches. Conversely, genetic analyses benefit substantially from integration with detailed osteological and archaeological information. Rather than replacing conventional anthropology, molecular approaches can strengthen it by providing independent lines of evidence and opportunities for methodological validation.

Taken together, the contributions to this Special Issue illustrate several broader developments currently reshaping population genetics and molecular anthropology. First, increasing genomic resolution is allowing population history to be reconstructed at progressively finer geographic and temporal scales. Second, aDNA has transformed population genetics from a discipline that largely inferred past demographic events from present-day variation into one capable of directly observing genetic variation at different points in time. Recent ancient genomic research increasingly moves beyond identifying major migrations and population replacements toward reconstructing kinship, social organization, adaptation, and interactions within individual communities [6,7].

Third, interdisciplinary integration is becoming central to the interpretation of genomic data. The papers assembled in this Special Issue illustrate this particularly well: genomic data are interpreted alongside historical evidence in medieval Catalonia, maternal and paternal lineages are contextualized within the complex demographic history of Southeastern Europe, and molecular sex determination is evaluated against traditional osteological approaches.

Nevertheless, substantial gaps remain. Geographic representation continues to be uneven, both in modern genomic databases and in the ancient DNA record. Some populations and regions are represented by thousands of genomes, whereas others remain characterized by small datasets or predominantly by uniparental markers. Temporal sampling is similarly heterogeneous, potentially creating artificial discontinuities in reconstructed population histories. Increasing the representation of understudied populations and archaeological periods will therefore be critical.

For aDNA research, continuing improvements in extraction, sequencing and computational analysis are expanding both the temporal range and the archaeological contexts that can be investigated. Ancient genomic research increasingly focuses less on broad continental patterns and more on specific communities and individuals [6]. Higher-resolution approaches to kinship, identity-by-descent, demographic modelling, and ancestry inference now provide opportunities to reconstruct family relationships, residence patterns, social organization, and population interactions. Ancient genomes can also provide information that cannot always be reliably reconstructed from contemporary populations alone. Selection signals, for example, may be obscured or modified in present-day genomes through subsequent admixture and genetic drift, whereas temporally stratified ancient genomes can permit direct investigation of allele-frequency changes through time [7].

Technological advances must be accompanied by continued attention to ethical practice. Human genomic data are inherently sensitive, and ancient human remains may have cultural, religious, or community significance extending far beyond their scientific value. Recent discussions have increasingly emphasized community engagement, relational approaches to consent, data governance, and the need to recognize the interests of living communities potentially connected to ancient individuals [8].

These considerations are especially important when research concerns historically identifiable groups, as demonstrated by the medieval Jewish population investigated by Pallarés-Viña et al. [4]. Questions surrounding ancestry, community identity, persecution, and cultural affiliation require particularly careful interpretation.

More broadly, molecular anthropology must avoid the simplistic equation of genetic ancestry with social, ethnic, linguistic, or national identity. Human populations are shaped by migration, mixing, isolation, demographic changes, culture, and history. Genetic similarity alone does not prove a shared cultural identity, and cultural or linguistic boundaries do not always match genetic ones. The Sekler study [1] illustrates why these dimensions should be investigated together while retaining their conceptual distinction.

When looking into the future, several directions emerge in the next phase of population genetics and molecular anthropology. Increasing global representation in genomic datasets remains a fundamental priority. Expanded sampling should include not only larger numbers of individuals but also populations, regions, and historical periods that remain underrepresented in existing databases. At the technological level, long-read sequencing, complete genome assemblies, and pangenome approaches should allow increasingly comprehensive characterization of human genomic diversity, including structural variants and genomic regions previously inaccessible to population-level analysis. For ancient populations, methodological improvements in DNA recovery and minimally destructive sampling will expand the archaeological material available for genomic investigation while helping preserve valuable anthropological collections. At the analytical level, increasingly sophisticated demographic modelling and artificial intelligence and machine-learning approaches offer substantial potential for identifying complex patterns in large genomic datasets and integrating genetic, phenotypic, environmental, and archaeological information. Their value, however, will depend on representative training data, transparent methodology, validation across populations, and interpretable models. Increasing computational power cannot compensate for biased population sampling or insufficient biological and historical context.

In conclusion, population genetics and molecular anthropology are increasingly converging toward a common objective: understanding human biological variation as a dynamic process unfolding across generations, populations, landscapes, and historical periods. The studies collected in “Emerging Topics in Population Genetics and Molecular Anthropology” reflect the continuing transition toward a more integrated understanding of human biological diversity; from Pleistocene hominin variability and medieval communities to the population histories of Southeastern and Central Europe and the molecular validation of anthropological methods, they demonstrate the broad range of research questions that can now be addressed through population genetics and molecular anthropology. At the same time, they show that technological sophistication is most informative when genetic evidence is interpreted alongside archaeological, morphological, historical, and demographic information. We hope that the contributions assembled in this Special Issue will stimulate further research at this intersection and encourage continued collaboration among geneticists, anthropologists, archaeologists, forensic scientists, historians, and researchers from related disciplines.
